# PEPOP 2.0: new approaches to mimic non-continuous epitopes

**DOI:** 10.1186/s12859-019-2867-5

**Published:** 2019-07-11

**Authors:** Vincent Demolombe, Alexandre G. de Brevern, Liza Felicori, Christophe NGuyen, Ricardo Andrez Machado de Avila, Lionel Valera, Bénédicte Jardin-Watelet, Géraldine Lavigne, Aurélien Lebreton, Franck Molina, Violaine Moreau

**Affiliations:** 1 BPMP, CNRS, INRA, Montpellier SupAgro, Univ Montpellier, Montpellier, France; 2 INSERM UMR-S 1134, DSIMB, F-75739 Paris, France; 3 Univ Paris Diderot, Sorbonne Paris Cité, Univ de la Réunion, Univ des Antilles, UMR 1134, F-75739 Paris, France; 4 INTS, F-75739 Paris, France; 5 Laboratoire d’Excellence GR-Ex, F75737 Paris, France; 6 Departamento de Bioquímica e Imunologia, Universidade Federal de Minas Gerais, Belo Horizonte, MG Brazil; 7 Sys2Diag UMR 9005 CNRS/ALCEDIAG, Complex System Modeling and Engineering for Diagnosis, Cap delta/Parc Euromédecine, 1682 rue de la Valsière CS 61003, 34184 Montpellier Cedex 4, France; 8 Programa de Pós-Graduação em Ciências da Saúde, Universidade do Extremo Sul Catarinense, Criciúma, Santa Catarina 88806-000 Brazil; 9 Bio-Rad Laboratories, 1682 Rue de la Valsière CS 61003, 34184 Montpellier CEDEX 04, France; 10 Department of Haematology, University Hospital, Nîmes, France; 11 Service d’hématologie biologique, CHU Clermont-Ferrand, Clermont-Ferrand, France; 12 Centre de Biochimie Structurale (CBS), INSERM, CNRS, Univ Montpellier, 29, route de Navacelles, 34090 Montpellier, France

**Keywords:** Peptide design, Discontinuous and continuous epitope, B-cell epitope, Ag-ab interaction, IPP, Protein surface, Structural bioinformatics, Immunogenicity, Antigenicity, Molecular mimicry

## Abstract

**Background:**

Bioinformatics methods are helpful to identify new molecules for diagnostic or therapeutic applications. For example, the use of peptides capable of mimicking binding sites has several benefits in replacing a protein which is difficult to produce, or toxic. Using peptides is less expensive. Peptides are easier to manipulate, and can be used as drugs. Continuous epitopes predicted by bioinformatics tools are commonly used and these sequential epitopes are used as is in further experiments. Numerous discontinuous epitope predictors have been developed but only two bioinformatics tools have been proposed so far to predict peptide sequences: Superficial and PEPOP 2.0. PEPOP 2.0 can generate series of peptide sequences that can replace continuous or discontinuous epitopes in their interaction with their cognate antibody.

**Results:**

We have developed an improved version of PEPOP (PEPOP 2.0) dedicated to answer to experimentalists’ need for a tool able to handle proteins and to turn them into peptides. The PEPOP 2.0 web site has been reorganized by peptide prediction category and is therefore better formulated to experimental designs. Since the first version of PEPOP, 32 new methods of peptide design were developed. In total, PEPOP 2.0 proposes 35 methods in which 34 deal specifically with discontinuous epitopes, the most represented epitope type in nature.

**Conclusion:**

Through the presentation of its user-friendly, well-structured new web site conceived in close proximity to experimentalists, we report original methods that show how PEPOP 2.0 can assist biologists in dealing with discontinuous epitopes.

**Electronic supplementary material:**

The online version of this article (10.1186/s12859-019-2867-5) contains supplementary material, which is available to authorized users.

## Background

The antigen-antibody (Ag-Ab) interaction is the basis of the immune system, and the Ab is a valuable tool in various biomedical applications, including diagnosis and therapy research [1, 2]. The Ab plays a key role in two phenomena: immunogenicity and antigenicity. Immunogenicity is the ability of a molecule to induce an immune response in the host, yielding Abs. Antigenicity is the ability of a molecule to bind specifically to an Ab. Abs are known to exhibit highly specific binding, though off-target binding can occur [3]. The paratope of the Ab interacts with the epitope of the protein Ag. An epitope can be continuous or discontinuous, linear or conformational [4–6]. A continuous, linear, or sequential, epitope is a fragment of the protein sequence. A discontinuous epitope is composed of several small fragments that are scattered in the protein sequence, but are close when the protein is structured. A conformational epitope has to be correctly structured to be recognized by the Ab and is often discontinuous, although it can also be continuous, for example, in the case of a constraint mimotope.

Epitope prediction tools have been developed for two major reasons [7, 8]. First, to identify in the protein fragments which are expected to be more efficient and specific than the rest of the protein in eliciting anti-protein Abs by immunization in a host. Second, to identify epitopes recognized by an existing Ab. These tools hope to overcome the difficulties in experimentally mapping epitopes on proteins [9, 10], as the most accurate method is the 3D structural identification of the Ag-Ab complex by X-ray crystallography, which is a time-consuming and laborious procedure.

The first epitope prediction tools predicted continuous epitopes from the protein sequence using propensity scales based on different physico-chemical properties [11] such as hydrophilicity [12], flexibility [13], β-turns [14], surface accessibility [15], or antigenicity [16]. Despite attempted improvements in the methodology [17, 18], among them the combination of properties [19], Blythe & Flower showed that the predictions are not better than chance [20]. It was supposed that because most of the epitopes are discontinuous [21, 22], the tools did not sufficiently take into account this criterion. The epitope prediction tools should consider structural information and target the identification of discontinuous epitopes. It is only rather belatedly that researchers have taken an interest in considering the 3D structure of the protein [23–25]. New epitope prediction tools are regularly developed [26–29].

Important research developments in this field do not concern real “ab initio” epitope prediction tools but fast and efficient methods dedicated to the complex task of dealing with discontinuous epitopes (either in helping to map them or in proposing immunogenic peptide sequences). These new bioinformatics methods could help in dealing with the discovery of new molecules, such as biomarkers or therapeutics, resulting from the high-throughput technologies like proteomics [30, 31]. They could provide solutions to characterize these new molecules by developing probes to capture them, by mapping epitopes, identifying interaction sites, finding peptide surrogates, etc. Despite the interest in using prediction tools, in the end, the experimentalist will use peptides, either for immunization or to replace the protein in the interaction with the Ab [32]. But, compared to continuous epitopes which are synthesized as is, the prediction of peptides mimicking discontinuous epitopes is more complicated as a correct arrangement between the elements composing the epitope has to be found in order to build the peptide (see Additional file 1). Moreover, it is known that the recognition of the Ab can be very sensitive to the sequence: only one mutation can alter the interaction (Duarte C et al., A mimic of a discontinuous epitope from AaH II identified by combining wet and dry experiments: a new experimental methodology to localize discontinuous epitopes, in preparation). Thus, using the relevant sequence is crucial. To date, only two bioinformatics tools propose the prediction of peptide sequences using 3D information: Superficial [33] and PEPOP [34]. Superficial predicts continuous and discontinuous peptides representing a potential epitope. The tool determines accessible protein fragments in a defined region on the protein and gathers them in a peptide, adding residues to link the fragments between them. PEPOP 2.0 is an antigenic and immunogenic peptide prediction tool. The first version of PEPOP proposed three different methods to design peptides and we showed that they can be used to generate anti-protein Abs [34] or to map epitopes [35]. In our new research, we have focused on novel methods that predict peptides representative of discontinuous epitopes and we have benchmarked them (Demolombe V et al., Benchmarking the PEPOP methods for mimicking discontinuous epitopes, submitted).

In this article, we present innovative methods, through different studies, which can bring solutions to biologists’ difficulties with discontinuous epitopes using PEPOP 2.0 and its new web site conceived in close proximity to experimentalists. Peptides predicted by PEPOP 2.0 have been used as immunogens to prepare anti-protein Abs using one peptide targeting one specific region. They have also been used in pairs to target two distinct regions on the protein, allowing the capture of the Ag. Peptides predicted by PEPOP 2.0 have then been used as Ags either to experimentally map an epitope or to find an inhibitor of an Ab-Ag interaction. We show the interest of using peptides that can represent the cognate protein. The ensemble of these improvements has been implemented in the improved web-site. PEPOP 2.0 is available at https://www.sys2diag.cnrs.fr/index.php?page=pepop.

## Results

### Description of PEPOP 2.0

PEPOP 2.0 [34] is a tool dedicated to the prediction of peptides able to replace a protein in its interaction with an Ab. PEPOP 2.0 computes different combinations between surface accessible segments or aa using 34 different algorithms (Experimental procedures, Table 1 and (Demolombe V et al., Benchmarking the PEPOP methods for mimicking discontinuous epitopes, submitted)) to finally propose one or a list of linear peptides mimicking discontinuous epitopes. A comparison of known epitopes [36] with PEPOP predictions is reported in Additional file 2: Table S1 and shows that PEPOP predictions can include on average 84% of the epitope aa.

**Table 1:** PEPOP 2.0 methods and their main characteristics

Category	Sub category	Name	Full name	Composing elements	Characteristic	Epitope type mimetic
	Sequential	FPS	Flanking Protein Sequence	protein sequence	Extension of a segment with the protein sequence	continuous
Nearest Neigbors	Prime methods	NN	Nearest Neighbors	segments in the natural orientation	Sequentially concatenation of NN segments	discontinuous
		uNN	upset NN	segments in the natural or reverse orientation	Sequentially concatenation of NN segments	
		FNN	Flanking NN	segments in the natural orientation	Concatenation in turn C- and N-terminally of NN segments	
		ONN	Optimized NN	segments in the natural orientation	Shortest path between the segments of NN method	
		OFN	Optimized Flanking NN	segments in the natural orientation	Shortest path between the segments of FNN peptides	
		OPP	Optimized Patched segments Path	segments in the natural orientation	Shortest path between the segments in a 10 Å-radius patch	
	Prime methods with ALA linker	NNala	NN with ALA linker	segments in the natural orientation	ALA linkers inserted between segments of NN method	
		uNNala	upset NN with ALA linker	segments in the natural orientation	ALA linkers inserted between segments of uNN method	
		ONNala	Optimized NN with ALA linker	segments in the natural orientation	ALA linkers inserted between segments of ONN method	
		FNala	Flanking NN with ALA linker	segments in the natural orientation	ALA linkers inserted between segments of FNN method	
		OFNala	Optimized Flanking NN with ALA linker	segments in the natural orientation	ALA linkers inserted between segments of OFN method	
		OPPala	Optimized Patched segments path with ALA linker	segments in the natural orientation	ALA linkers inserted between segments of OPP method	
	Prime methods with structural-based linker	NNsa	NN with SA linker	segments in the natural orientation	Linkers computed from SA inserted between segments of NN method	
		ONNsa	Optimized NN with SA linker	segments in the natural orientation	Linkers computed from SA inserted between segments of ONN method	
		FNsa	Flanking NN with SA linker	segments in the natural orientation	Linkers computed from SA inserted between segments of FNN method	
		OFNsa	Optimized Flanking NN with SA linker	segments in the natural orientation	Linkers computed from SA inserted between segments of OFN method	
		OPPsa	Optimized Patched segments Path with SA linker	segments in the natural orientation	Linkers computed from SA inserted between segments of OPP method	
	Prime methods with superposed structural-based linker	NNsas	NN with SAS linker	segments in the natural orientation	Linkers computed from SAS inserted between segments of NN method	
		ONNsas	Optimized NN with SAS linker	segments in the natural orientation	Linkers computed from SAS inserted between segments of ONN method	
		FNsas	Flanking NN with SAS linker	segments in the natural orientation	Linkers computed from SAS inserted between segments of FNN method	
		OFNsas	Optimized Flanking NN with SAS linker	segments in the natural orientation	Linkers computed from SAS inserted between segments of OFN method	
		OPPsas	Optimized Patched segments path with SAS linker	segments in the natural orientation	Linkers computed from SAS inserted between segments of OPP method	
Graph Theory	SHP methods	SHPnat	SHP natural	segments in the natural orientation	Shortest path between segments using Dijkstra’s algorithm	
		SHPrev	SHP reverse	segments in the natural or reverse orientation	Shortest path between segments using Dijkstra’s algorithm	
		SHPaa	SHP amino acids	amino acids	Shortest path between segments using Dijkstra’s algorithm	
	TSP methods	TSPnat1	TSP natural 1	segments in the natural orientation	Shortest path between segments using Dantzig & Fulkerson’s algorithm and most favorable interacting parameters	
		TSPnat2	TSP natural 2	segments in the natural orientation	Shortest path between segments using Dantzig & Fulkerson’s algorithm	
		TSPnat3	TSP natural 3	segments in the natural orientation	Shortest path using Dantzig & Fulkerson’s algorithm according to the number of segments	
		TSPnat4	TSP natural 4	segments in the natural orientation	Shortest path using Dantzig & Fulkerson’s algorithm including the 2 closest segments	
		TSPrev1	TSP reverse 1	segments in the natural or reverse orientation	Shortest path using Dantzig & Fulkerson’s algorithm and most favorable interacting parameters	
		TSPrev2	TSP reverse 2	segments in the natural or reverse orientation	Shortest path using Dantzig & Fulkerson’s algorithm	
		TSPrev3	TSP reverse 3	segments in the natural or reverse orientation	Shortest path using Dantzig & Fulkerson’s algorithm according to the number of segments	
		TSPrev4	TSP reverse 4	segments in the natural or reverse orientation	Shortest path using Dantzig & Fulkerson’s algorithm including the 2 closest segments	
		TSPaa	TSP amino acids	amino acids	Shortest path using Dantzig & Fulkerson’s algorithm	

*ALA* alanine, *NN* nearest neighbor, *SA* structural alphabet, *SAS* superposed structural alphabet, *SHP* SHortest Path algorithm, *TSP* Traveling Salesman Problem algorithm

**Fig. 1:**
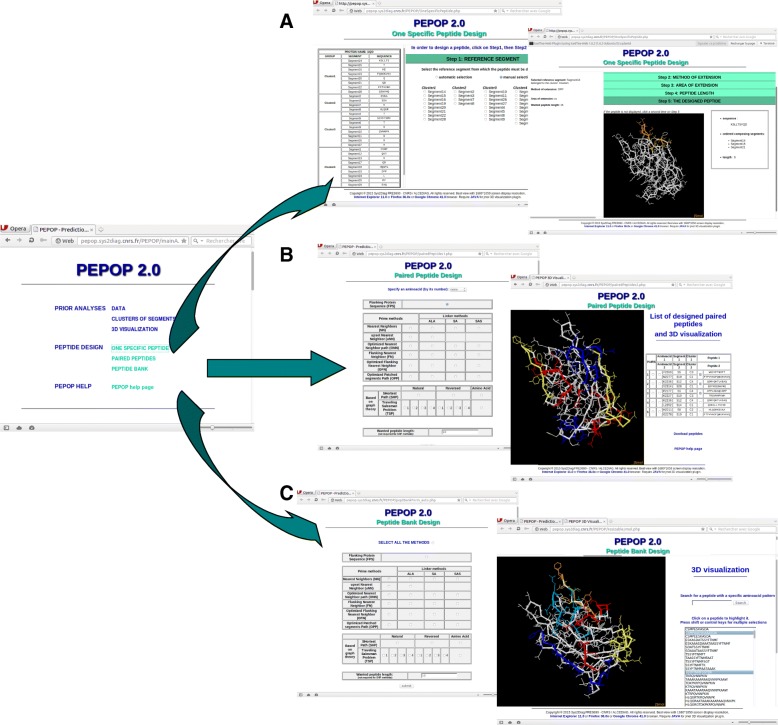
PEPOP 2.0 web-site. The first result page of PEPOP 2.0, after the user gives the 3D structure of the protein, proposes 3 different ways to design peptides. **a** The ‘One Specific Peptide Design’ predicts one peptide at a time through 5 steps where the user has to select the reference segment (first insert), the method of extension, the area of extension and the peptide length; the fifth step (second insert) gives the peptide sequence and displays it on the 3D structure of the protein. **b** To design peptides in the ‘Paired Peptide Design’ section, the user selects the method of extension, the peptide length and eventually the aa from which the first pair has to be determined (first insert); the 5 peptide pairs are summarized in one side of the browser and displayed on the 3D structure of the protein on the other side of the browser. **c** In the ‘Peptide Bank Design’, the user has to select the method(s) and the peptide length (first insert); all the predicted peptides can be displayed on the 3D structure of the protein (second insert)

PEPOP 2.0 is available in an improved new version of the web site (Fig. 1). The web interface is composed of 3 sections that can correspond to different ways to use PEPOP 2.0 in experimental projects. Below are four examples using PEPOP 2.0 to predict peptides and use them in experiments. Each user is free to imagine other ways to use these “discontinuous” peptides.

The sections ‘One Specific Peptide Design’ and ‘Paired Peptide Design’ are dedicated to the prediction of peptides that will be used to generate anti-protein Abs. The ‘Peptide Bank Design’ section of the PEPOP 2.0 web site is dedicated to the design of peptides that will be used for their antigenic properties. For this section, two types of experiments have been illustrated: the mapping of discontinuous epitopes and the identification of inhibitor peptides.

### Designing peptides to generate anti-protein abs

The ‘One Specific Peptide Design’ section of the PEPOP 2.0 web site is dedicated to the prediction of one peptide at a time. This section already existed in the previous version of PEPOP 2.0 but was updated and enriched with new methods. This section allows defining only a small number of peptides. The peptide is progressively built through 4 steps: the reference segment, the method of extension, the area of extension and the peptide length. At each step, a choice is selected by default so that at the end the peptide can be built automatically. Instead, the user may control the choices and the parameters (the 5 physicochemical and structural criteria: hydrophobicity, accessibility, segment length, β-turn content, WRYP content) at any step.

**Fig. 2:**
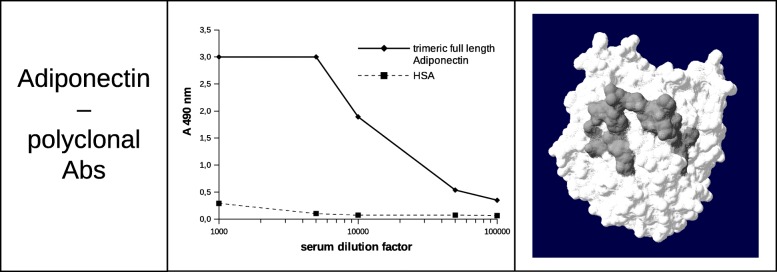
Reactivity of mouse immune serum raised using a “discontinuous” peptide against trimeric full length adiponectin (LMW adiponectin) and a control protein (HSA). The segments composing the peptide are displayed on the surface of the protein

Using this section of PEPOP, we designed a peptide from the 3D structure of the LMW (low molecular weight) form of adiponectin (PDB code: 1C3H). The peptide KYGDGDHNGLYADVETR has been predicted by the OFN method and gathered 4 segments: sequentially, segment 70 (K), segment 80 (YGDGDHNGLYAD), segment 81 (V), and segment 58 (ETR). The OFN method adds the sequence of the nearest neighbor segment C-terminally and then N-terminally until the requested length of the peptide is reached. We chose this method, new in this version of PEPOP 2.0, because we think it could be important to keep the reference segment in a central position in the peptide to be more easily recognized by the Ab. After peptide mouse immunization, we observed that Abs against the predicted “discontinuous” adiponectin peptide were able to recognize the trimeric full-length adiponectin but did not recognize the human serum albumin (Fig. 2). The representation of the segments on the 3D structure of the protein clearly shows that despite the fact that they are not contiguous on the sequence, they are gathered in one region of the protein (Fig. 2). This result showed that PEPOP 2.0 successfully designs a peptide able to generate Abs targeting a discontinuous epitope on the cognate Ag.

### Designing peptides to generate abs capturing the cognate protein

**Fig. 3:**
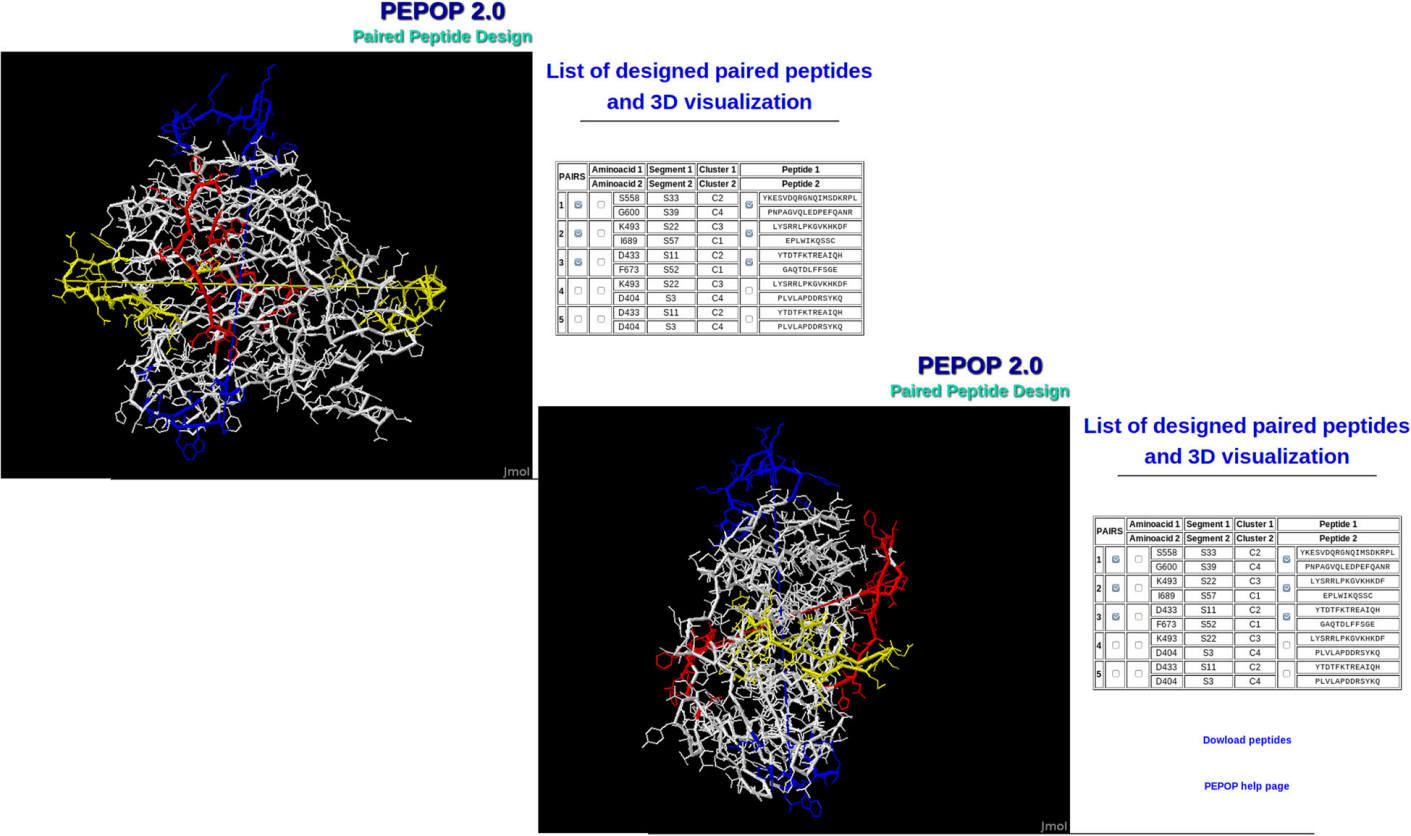
Example of paired predicted peptides on the A2 domain of FVIII. Paired peptides have been predicted from two distinct regions on the A2 domain of FVIII. The 6 peptides are in distinct and opposite (two by two) regions of the protein. The first paired peptides is in yellow, the second in blue and the third in red. The two 3D structure views are orthogonal

The ‘Paired Peptides Design’ section is new in this improved version of PEPOP 2.0. It is dedicated to the prediction of pairs of peptides. The goal is to target specific and distinct regions on the protein: the predicted peptides can then be used to prepare Abs that should be able to capture the cognate protein. The principle is to select two candidate peptides that are appropriately structurally separated in the 3D model. PEPOP 2.0 proposes up to 5 pairs of distinct peptides. The peptides are designed by computing the most distant pairs of surface accessible aa and the two orthogonal most distant pairs in order to give the best chance for the generated Abs to capture the Ag without steric hindrance. Two more pairs are proposed as an alternative in the event that a targeted region is too close to the first one. This would lead to steric hindrance for the Abs generated. The user can orientate the design by indicating the position of one of the two aa of the first pair. The other pairs will be designed consequently. Figure 3 shows an example of the three first paired peptides on the A2 domain of FVIII. The six peptides are in distinct and opposite (two by two) regions of the protein. The recognition of the protein by the Abs generated by such peptides should not be disturbed by steric hindrance. The Abs should capture the protein two by two. This section of PEPOP 2.0 can be a useful tool for the characterization of the proteins after a process of high throughput selection or for the development of a kit for diagnosis.

We showed how PEPOP 2.0 can propose peptides to use in immunogenic experiments. The designed peptides can also be used for their antigenic properties.

### Designing peptides to map discontinuous epitopes

The new section ‘Peptide Bank Design’ has been designed to propose an alternative to the existing time- and ressource-consuming methods used to map discontinuous epitopes. The idea is to use a mixture of experiments to map continuous (high-throughput peptide synthesis, e.g. SPOT technology [37, 38]) and discontinuous epitopes (e.g. phage-display). As all the epitope information is already contained on the protein, experimental design is best suited by only testing the most numerous possible peptides, as in phage-display experiment. We drastically reduced the peptide search space by using protein information and methods carefully considered to address antigenic properties. The virtual peptide sequence bank is constructed thanks to a flexible web interface where the user has to choose the methods of extension and the peptide length (set to 10 aa length by default). Each method predicts all the possible peptides. For example, in the case of the prime, ALA, SA, and SAS methods, all the segments determined by PEPOP 2.0 are individually selected as the reference segment. Thus, the method predicts as many peptides as segments. In this way, the entire surface of the protein is explored. Moreover, using several methods allows the testing of different arrangements of the same segments in peptides. Indeed, as we do not really know what governs the antigenic rules, we do not really know how some peptide characteristics, such as the peptide conformation, the aa position, the aa spacing, or the aa order influence the interaction with the Ab. The predicted peptides can be visualized on the 3D structure of the protein one or several at a time.

**Fig. 4:**
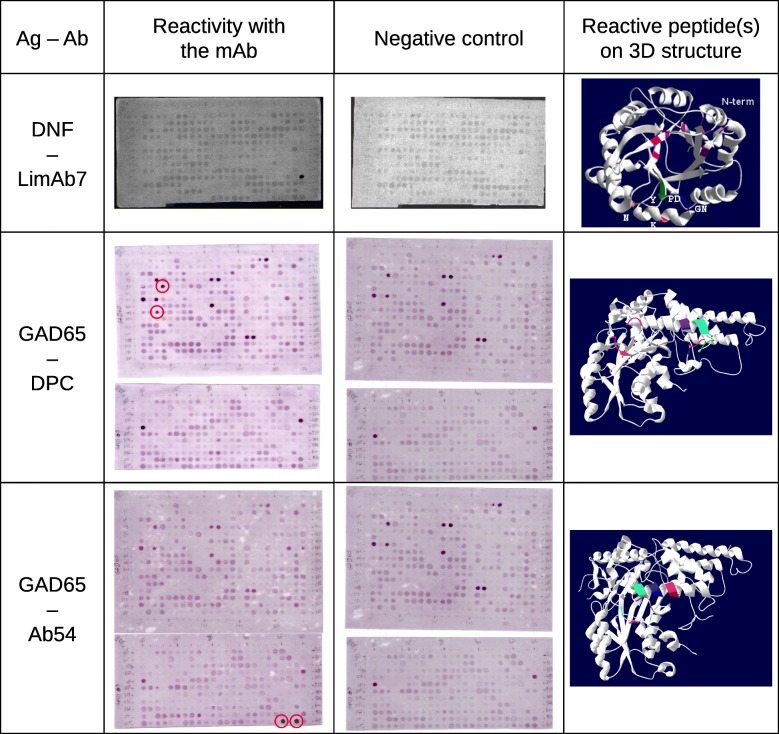
Reactivity of monoclonal antibodies, LimAb7, DPC and GAD65 with “discontinuous” peptides predicted from the 3D structure of respectively LiD1 and GAD65. The peptides have been prepared by the Spot technology. The reactivity was controlled with anti-Fc pAbs alone. The reactive peptides with the mAb are displayed on the 3D structure of the corresponding protein

Using this methodology we map discontinuous epitopes either recognized by a pAbs on Amm8 [35] or recognized by mAbs on AaH II (Duarte C et al., A mimic of a discontinuous epitope from AaH II identified by combining wet and dry experiments: a new experimental methodology to localize discontinuous epitopes, in preparation) and GM-CSF (Abraham J-D et al., Combination of bioinformatics and experimental approaches to map the conformational epitope on GM-CSF, in preparation). Figure 4 shows three more studies mapping discontinuous epitopes on LiD1 recognized by LimAb7 mAb [39, 40] and GAD65 recognized by DPC mAb and Ab54 mAb. Using prime, ALA, and SA methods with a requested peptide length of 10 aa, 456, and 648 peptides were predicted from the 3D model of LiD1 [41] and the 3D structure of GAD65 (PDB code: 2OKK) respectively. Peptides shorter than 7 aa have been eliminated because it is considered that the peptide is too short to well mimic the discontinuous epitope. Peptides longer than 24 aa have been eliminated due to synthesis performance limitations. Peptides have been synthesized using the SPOT method and their immune reactivities were tested with their respective mAb. In the case of LiD1, only one peptide has been recognized: it is displayed on the 3D structure of the protein. For GAD epitopes, several peptides have been identified. However, the control experiment with only anti-Fc pAbs reveals the reactivity of several peptides. By subtracting them, two specific spots appear that are only recognized by the mAb. According to the mAb, either DPC or Ab54, the two spots are different. The peptides representative of discontinuous epitopes are displayed on the 3D structure of GAD65. These results, with previous studies [35] (Duarte C et al., A mimic of a discontinuous epitope from AaH II identified by combining wet and dry experiments: a new experimental methodology to localize discontinuous epitopes, in preparation; Abraham J-D et al., Combination of bioinformatics and experimental approaches to map the conformational epitope on GM-CSF, in preparation), showed that PEPOP 2.0 successfully designs “discontinuous” peptides able to be recognized by the Abs allowing the localization of the targeting discontinuous epitopes on the cognate Ag.

### Designing peptides to identify inhibitor peptides

**Fig. 5:**
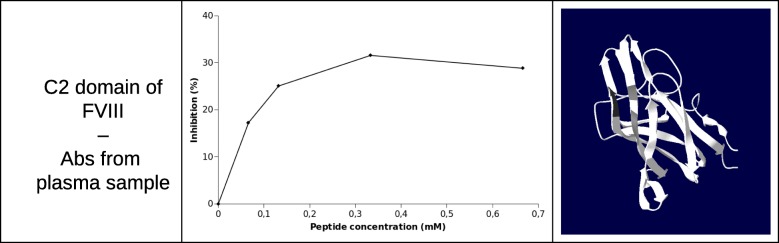
Inhibition obtained with different amounts of a peptide representative of the C2 domain of FVIII in x-MAP inhibition assays using plasma sample

Another way to use the ‘Peptide Bank Design’ section of the PEPOP 2.0 web site is to test the antigenicity of the predicted peptides synthesized in soluble form with Abs in order to select peptides that could replace the cognate protein. Prediction of epitopes could have potential clinical implications in hemophilia A (HA), an inherited bleeding disorder. Indeed, severe HA is defined by an undetectable level of coagulation factor VIII (FVIII). The treatment of HA is based on regular intravenous infusions of FVIII and, to date, the main complication (up to 30% of severe HA patients) of this treatment is the development of inhibitory anti-FVIII Abs. The development of this immune response dramatically impacts the care of HA patients, and a fine epitope mapping could be helpful for a better understanding of the physiopathology and the treatment of such complications. As anti-FVIII Abs are mainly directed against C2 and A2 domain of FVIII, we predicted peptides mimicking discontinuous epitopes of these domains [42, 43]. For example, we synthesized 33 synthetic peptides potentially representative of discontinuous epitopes on the C2 domain of coagulation FVIII, using the OPP method of PEPOP 2.0 [42]. Only one method has been selected in the ‘Peptide Bank Design’ section. As the experiments are relatively costly (in time and money) and need a large amount of plasma, all the peptides from the methods cannot be tested and a limited number of peptides needed to be selected. One solution is to select only one method. We chose this method because the reference segment is central in the patch, it contains no aa linker which could interfere with the Ab binding, and the search of the path between the segments is optimized. In this way, the peptides together still allow exploring the entire surface of the protein. Using an inhibition assay based on the x-MAP technology, we evaluated their ability to block the binding to the C2 domain of anti-C2 domain Abs from plasma samples. Figure 5 shows one of the reactive peptides blocking the Ab binding in a dose-dependent manner. The peptides inhibit the interaction between the C2 domain of FVIII and the Abs by around 30%. The same protocol with another PEPOP method, TSPaa, was used to predict peptides mimicking discontinuous epitopes of the A2 domain of FVIII [43]. So, we show that it is possible to find at least one peptide in a series predicted by PEPOP 2.0 that inhibits an Ab-Ag interaction. These results showed that PEPOP 2.0 successfully designs “discontinuous” peptides able to be recognized by the Abs targeting the cognate Ag.

For all sections of the PEPOP 2.0 web site, the location of the predicted peptides can be displayed on the 3D structure of the protein.

## Discussion

By presenting the improved version of the PEPOP 2.0 web-site, we showed the ways to use predicted peptides expected to mimic discontinuous epitopes. The most often use of the peptides is the generation of anti-protein Abs. One of the two great novelties of PEPOP 2.0 is the use of peptides by pair so as to target distinct regions on the surface of the protein and generate Abs that should be able to capture the protein. This can be a useful tool, for example, in the characterization of biomarkers after the process of discovery in high-throughput selection. Notably, it could lead to the development of diagnosis kits. The other novel feature of PEPOP 2.0 is the ‘Peptide Bank Design’ section of the web-site. Because we predict from the native Ag, we showed that only a limited number of peptides (compared to the diversity generated in phage-display method) is necessary to map discontinuous epitopes. After synthesis, the functionality of the peptides exploring all the surface of the protein could be assessed in a convenient high-throughput recognition assay, such as the SPOT method [35, 44, 45] or other technologies [46]. If the correct sequence is present in the bank, the Ab should recognize it and this identifies the epitope region on the protein. Then, a set of peptides around the space of the epitope region identified can be tested in further experiments to more precisely hone the epitope or to select a functional peptide. The final feature we tested is the search for an inhibitor. We synthesized, in soluble form, a restricted list of peptides and tested their capacity to inhibit the interaction between the protein and Abs. We showed that it is possible to select peptides able to replace discontinuous epitopes in an Ag-Ab interaction.

Two opposing views exist about epitopes. The first view considers that a protein is constituted by a mosaic of overlapping epitopes [47, 48]. It is therefore theoretically possible to generate Abs against any region of the protein surface. Specific phenomena such as, for example, central and peripheral immunotolerance [49], repetitive fragments [50] or aggregates [51] can induce variations in the immune response. However, using different hosts or different techniques [52–56] would allow the systematic acquisition of Abs. Any region on a protein is a potential epitope. The other point-of-view considers that proteins have only a few epitopes preferentially recognized by the immune system [57, 58]. In view of these two hypotheses, it is not surprising that Blythe and Flower found that the continuous epitope prediction tools are not better than chance [20] and that the discontinuous epitope prediction tools showed weak performances [36]. In the first hypothesis, a tool cannot find any region emerging from the others since it is possible to produce Abs targeting any surface of the protein. In the other hypothesis, it would likely be logistically impossible for a tool to well predict when the learning data are a mix of a variety of different epitopes (immunogen, epitopes generated from peptides, truncated protein, cross-reacting molecules) [59]. Theoretically, a tool cannot predict an epitope because an epitope only exists thanks to the existence of the Ab recognizing it. To know whether it is really possible to predict epitopes ab initio, the existence of immunodominant regions should be proved or refuted, for example with systematic studies by categorizing Ag-Ab complexes, distinguishing epitope types and origins. Perhaps, we will discover that it is an intermediary or both of the two hypotheses: the immune system could preferentially target few specific regions on the protein (would it be just a question of surface accessibility?) but it is still possible to produce Abs targeting any regions [60, 61]. Whatever the reality, in the present state of knowledge, the only way to predict an epitope is to take into account the Ab [62].

Predicting an epitope begins by proposing a region on the protein, i.e. a set of aa. Peptide prediction tools have to determine the sequence from this set by determining an arrangement, a disposition, a path between the aa. This can be very difficult. More elements have to be combined, and as the problem becomes more complex, it becomes rapidly unsolvable. This is an NP-complex problem relying on combinatorial mathematics. Solutions have to be found because it is impossible to enumerate all the possibilities.

Moreover, although the Ag-Ab interactions have been deeply studied [63–66], the mimicking of a discontinuous epitope by a linear peptide is still a challenging task [67]. Other parameters than those found in protein-protein interface studies [68–70] have to be taken into account. Should the peptide adopt the same conformation as in the protein so the Ab can recognize it? Would the peptide be in the same conformation in the protein context? Chen et al. [65] showed that the conformations of the peptides compared to those of the corresponding regions on the proteins when complexed with the Ab have considerable differences. It should be even more difficult because the structure of an epitope when it is complexed with the mAb tends to differ from the structure before the mutual adaptation process [71]. Should the aa be spaced out as in the protein so that they are correctly laid out to allow the CDR loops of the Ab to properly face and interact with them? Or, is it sufficient for the key aa to be present in the peptide whatever their disposition? In reality, molecular mimetism is poorly understood. It would be very informative to carry out systematic studies in order to fully elucidate this phenomenon. In this way, PEPOP 2.0 can be seen as a “test tube” to help to better understand molecular mimicry.

As molecular mimicry is still poorly understood, it is difficult to predict which peptide compared to another will be recognized by a specific Ab even if they are both composed of the same key aa. Consequently, it might be considered whether a scoring function is conceivable. We have deliberately chosen not to rank peptides: we would not know which rules are really important. Moreover, we think bioinformatics predictions cannot be used as such and have to be always associated to experiments. Combining bioinformatics predictions and simple experimental methods can be an interesting alternative to expensive and time-consuming approaches. The section “Peptide Bank Design” has been developed in the idea that it can be used in epitope mapping by associating it with SPOT methods. Somehow, the experiment replaces the scoring function: for a reduced time and cost, a more confident result is gained.

Moreover, there is a real advantage in using mimicking peptides. Beyond avoiding the difficulties of obtaining a pure preparation of the protein, reduction in cost, and increased ease in manipulation, even with polyclonal Abs the regions targeted on proteins are well known. The main advantage of using “discontinuous” peptides is that the final Abs should recognize the native well-structured protein Ag. Moreover, the same series of peptides can be probed by different Abs raised against the same target Ag, so as to disclose the cognate epitope of each.

However, the experimentalists have to carefully think through their experiments before designing peptides because, as van Regenmortel underlined at a workshop about the current state and future directions for the epitope prediction field [72], the results can be different according to the experiment. For example, a peptide seen reactive in SPOT could be found not interacting in the soluble form in ELISA. It may be due to the different conformation the peptide adopts according to whether it is linked to a support or totally free in solution. It also may be due to the phenomenon of avidity in SPOT. Thus, if the experimentalist wants to map the epitope, (s)he can carry out SPOT experiments or other high-throughput technologies. But, if (s)he wants to use the reactive peptide in further experiments, (s)he has to keep in mind that they may not react the same way. This is why it is recommended for the experimentalist searching for an inhibitory peptide to select it by using technology that will present the peptides in its final format. Furthermore, the experimentalist also has to carefully choose the peptide design methods according to the objectives of the experiment. If the aim is to generate Abs, it would be better not to use linker methods in order to avoid Abs that are directed against the linker aa, which could lead to Abs not cross-reacting with the protein. If the aim is to find an inhibitory peptide (experiments in soluble form), it is recommended to use peptide design methods that search for an optimized path (ONN, OFN, OPP, graph-based methods).

## Conclusion

Assigning a function to each new protein structure resulting from high-throughput genomics experiments is a huge task. For example, the current techniques for epitope mapping are unfeasible on a genomic scale due to the high cost and effort needed. Reliable computational methods can assist by offering fast, scalable, and cost-effective approaches for identifying B-cell epitopes, focusing on experimental studies and improving our understanding of Ag-Ab interactions. In silico tools such as PEPOP 2.0, designing immunogenic or antigenic peptides representative of a given protein should help experimentalists to handle proteins by turning them into peptides, which are smaller and easier to manipulate. They can aid with future goals in the area of the discovery of biomarkers by providing solutions to characterize these molecules or develop probes to capture them, leading to diagnosis and therapy applications. PEPOP 2.0, and its potential counterparts, will also be useful tools to discover and study the rules governing molecular mimicry by testing the different approaches developed in the peptide design methods through systematic studies on antigenicity or immunogenicity. The flexibility of PEPOP 2.0 allows other problems to be addressed. For example, one can compare the peptides representing the surface of two proteins known to interact with the same mAb. Or, as PEPOP 2.0 explores the surface of any protein, it can potentially be used to investigate any protein-protein interaction: the Ab would be replaced by another protein interacting with a targeted protein. Therefore, the protein-protein interaction site or an inhibitory peptide could be searched for in the same way. This opens the door to an even greater world of possibilities in diagnosis or therapy applications.

## Methods

### PEPOP 2.0

#### Principle

To predict a peptide, the first step is the computing of the surface accessible amino acids (aa) using default parameters of DSSP [38]. Continuous segments are then deduced. The second step is the determination of the segment or aa combination which will form the final peptide. To assemble the segments or the aa in an arrangement the Ab will be able to recognize, thirty-four methods can be used. They are based on different algorithms (for more details see Table 1 and (Demolombe V et al., Benchmarking the PEPOP methods for mimicking discontinuous epitopes, submitted)). The segments or aa used to build the final peptide come from a defined area of about an epitope size on the protein. The possible areas in PEPOP 2.0 can be either clusters of clustered segments according to their spatial distances or patches of 10 Å, 15 Å, and varying radii.

#### Patch definitions

A patch gathers the segments or aa present inside a radius from the G point of a reference (segment or aa, selected by the user or by default) to a defined distance. PEPOP 2.0 uses three types of patches. The 10 Å- and 15 Å-radius patches gather the segments in the fixed distance of 10 Å and 15 Å respectively. In the varying patch, the patch gathers the aa in the distances varying from 15 to 20 Å: the final radius corresponds to the one for which the number of aa collected is the average number of aa between radius 15, 16, 17, 18, 19 and 20 Å.

#### Prediction of paired peptides

The first step is to determine the distant aa. Then, the peptides are designed according to the method either by considering the aa or the segment including it as the reference (starting point).

The first pair of distant aa is the two most distant surface accessible aa of the protein. To find the second pair of aa, an orthogonal plane to the first pair of aa is drawn. The two most distant aa around 5 Å from this plane are sought. A distance from the plane has to be tolerated, otherwise the plane could cross a zero aa threshold. The third pair is the two most distant aa included in the 10 Å-thickness perpendicular bisector to the first and the second pairs of aa. The fourth and fifth pairs are proposed as an alternative to the second and third pair, respectively. The fourth pair of aa consists of one of the two aa of the second pair and the most distance of all the surface-accessible aa of the protein. The fifth pair of aa is the most distant pair where one is one of the two aa of the third pair and the other is found among all the surface-accessible aa of the protein.

#### Web interface

PEPOP 2.0 has been implemented on a virtualized Linux server kernel 2.6 running the Apache web server version 2.2.15. The tool has been implemented in object-oriented PHP and JavaScript, and uses scripts and software developed in PERL, C, and C++. Segments, clusters, and peptides identified by PEPOP 2.0 can be directly visualized on the 3D structure of the Ag thanks to jsmol. PEPOP 2.0 is available at https://www.sys2diag.cnrs.fr/index.php?page=pepop.

### Experiments

#### Synthesis of spot peptides

The peptide analogs were prepared by Spot synthesis [73] on a cellulose membrane, as previously described by Laune et al. [74]. Membranes were obtained from Proteigene. A Multipep robot (Intavis) was used for the coupling steps. Peptides were acetylated at the N-terminus. After the peptide sequences were assembled, the side-chain protecting groups were removed by trifluoroacetic acid treatment, but peptides remained attached on the membrane for ELISA-Spot experiments. Briefly, after an overnight saturation step with 3% BSA, the set of membrane bound peptides were probed by incubation with the mAb of interest. After 90 min incubation at room temperature, the membrane was washed and incubated for 1 h at room temperature with a peroxidase-conjugated anti-mouse or anti-human Ab (Sigma, diluted 1:3000). The spots were stained with enhanced chemiluminescent ECL detection kit (Amersham). The reactivity of each membrane was assessed in at least three independent experiments.

#### Synthesis of the “discontinuous” adiponectin soluble peptide and the coagulation FVIII soluble peptides

The soluble peptides were synthesized on a Multipep Synthesizer using fluorenylmethyloxycarbonyl (Fmoc) acting as a protective group [75, 76] with a HOBt-DIPC protocol. The C-terminal residues were first fixed to the solid phase support and NH2 extremity, and R groups are initially protected by Fmoc. After a basic deprotection of the NH2 extremity of the first fixed aa, the second protected aa was added and its carboxyl function activated to allow for the peptide linkage and extension of the peptides. The peptides were elongated after a succession of protection/deprotection steps until the addition of the last residue. Lateral chains were subsequently deprotected and the peptides released from the resin by trifluoroacetic acid treatment in the presence of the appropriate scavengers in order to generate amidated peptides. After synthesis, the peptides were lyophilized and the quality of the peptides was verified by high performance liquid chromatography and mass spectrometry.

#### Immune response to the “discontinuous” adiponectin peptide

##### Mouse immunization

Eight-week-old Balb/C male mice from Harlan (Gannat, France) were immunized with KLH-conjugated discontinuous adiponectin peptide. Injections were performed every 2 weeks with 20 μg of KLH-peptides emulsified in complete Freund adjuvant (first injection) or incomplete Freund adjuvant (following injections). The 2 first injections were performed via intraperitoneal route and the following injections via subcutaneous route. After the 5th injection, blood was collected in order to characterize the immune response to full-length adiponectin. The study was approved by the “direction départementale de la protection des populations” (B34–172-27 agreement).

##### Immune response characterization

Binding activity of the immune serum to recombinant adiponectin and irrelevant protein was evaluated by direct ELISA. The plates were coated overnight at 4 °C with 1 μg/ml of recombinant trimeric form of human adiponectin produced in HEK cells (BioVendor, #RD172091100) or purified Human Serum Albumin (HSA) (Sigma-Aldrich, A9511). After blocking with 1% milk in PBS, mouse serum was diluted from 1/1000 to 1/100000 in PBS with 0.1% milk and 0.1% Tween and plates were incubated for 1 h. After washing, the secondary Ab Peroxidase-AffiniPure Donkey Anti-Mouse IgG (Jackson Immuniresearch, 715–035-150) was incubated for 1 h at 1/3000 in the same buffer followed by o-Phenylenediamine dihydrochloride (Sigma Aldrich, P8412). The absorbance was measured at 490 nm.

#### Plasma reactivity with the discontinuous peptides from coagulation FVIII

The ability of peptides to inhibit the binding of anti-C2 Abs to the C2 domain was then evaluated in an original and homemade inhibition assay. Briefly, the domain of interest was immobilized on luminex beads. The plasma of hemophilia A (HA) patients containing anti-C2 Abs was incubated with a range of concentrations of peptides and thereafter incubated with beads. The dose-dependent inhibition of peptides was revealed with a fluorescent anti-human Ab, recognizing residual plasma Abs bound to the specific domain coated on beads. If the predicted peptide mimicked one of the epitopes recognized by human anti-C2 Abs, the level of fluorescence decreased and the inhibition rate increased. This study was connected to Lapalud et al. study [77] for which the protocol was approved by the Ethics Committee of Montpellier (France), and informed consent was obtained for all patients in accordance with the Declaration of Helsinki.

## Additional files

Additional file 1: **Sup data 1.** Description of the NP-problem when predicting a peptide expected to mimic a discontinuous epitope (PDF 36 kb)

Additional file 2: **Table S1.** Comparison of 75 known epitopes with PEPOP predictions. (XLSX 12 kb)

## Abbreviations

aa: Amino acids
Ab: Antibody
Ag: Antigen
FVIII: Factor VIII
HA: Hemophilia A
