# Supplementary material for: PEPOP 2.0: new approaches to mimic non-continuous epitopes
Source: BMC Bioinformatics. 2019 Jul 11;20:387. doi: 10.1186/s12859-019-2867-5 (PMC6625012; doi:10.1186/s12859-019-2867-5)
Supplement: Supplementary file 1 — Sup data 1. Description of the NP-problem when predicting a peptide expected to mimic a discontinuous epitope (PDF 36 kb) [file 12859_2019_2867_MOESM1_ESM.pdf]

Supplementary data

sup data 1

When the tools predicted continuous epitopes, the peptide is ready to be synthesized and used. However, the tools based on the 3D structure of the protein generally predicted regions on the protein and listed the aa potentially being in the epitope. But, among these aa, how determine the sequence of the peptide to use in experiments? Enumerating all the possible sequences comes under combinatorics and is a NP-complete problem in mathematics. To give an idea, the number of sequences of 10 aa or less, dipping into the 20 aa, are 10,778,947,368,420 (

$\frac{N(N^L - 1)}{(N-1)}$, with L the highest length and N the number of elements, here the 20 aa).

More concretely, all the possible sequences formed with 15 defined aa is equal to 15! (1307674368000 sequences). It would be impossible to test all the possible peptides experimentally or even computationally, so a limited enumeration must be defined.
